# BTK, the new kid on the (oncology) block?

**DOI:** 10.3389/fonc.2022.944538

**Published:** 2022-08-04

**Authors:** Emanuela Grassilli, Maria Grazia Cerrito, Marialuisa Lavitrano

**Affiliations:** Laboratory of Molecular Medicine, Department of Medicine and Surgery, University of Milano-Bicocca, Monza, Italy

**Keywords:** BTK, BTK-A, BTK-C, p65BTK, B-cell leukemia, solid tumors

## Abstract

In the last decade data piled up indicating that BTK – for twenty years considered as a “private matter” of bone marrow-derived cells – it is expressed and plays important and different roles also outside of the hematopoietic compartment and, most notably, in tumor cells. Initial evidence that BTK plays a critical role in B cell-derived malignancies prompted the chase for specific inhibitors, the forefather of which entered the clinic in a record time and paved the way for an ever increasing number of new molecules to be trialed. The growing interests in BTK also led to the discovery that, in solid tumors, two novel isoforms are mainly expressed and actionable liabilities for target therapy. Remarkably, the different isoforms appear to be involved in different signaling pathways which will have to be attentively specified in order to define the area of therapeutic intervention. In this perspective we briefly summarize the progress made in the last decade in studying BTK and its isoforms in cancer cells and define the open questions to be addressed in order to get the most benefits from its targeting for therapeutic purposes.

## Introduction

The discovery of the Bruton tyrosine kinase (BTK) as the product of the defective gene in X-linked agammaglobulinemia goes back to almost thirty years ago (1). However, the further we go in studying this kinase, the more facets we discover in a protein that once was believed to be one size-fits-it all and hematopoietic-specific. For this reason, BTK has been gaining tremendous momentum in recent years, taking center stage in different biological scenarios and becoming one of the “hottest” actionable targets in several diseases, including cancer.

**Figure 1:**
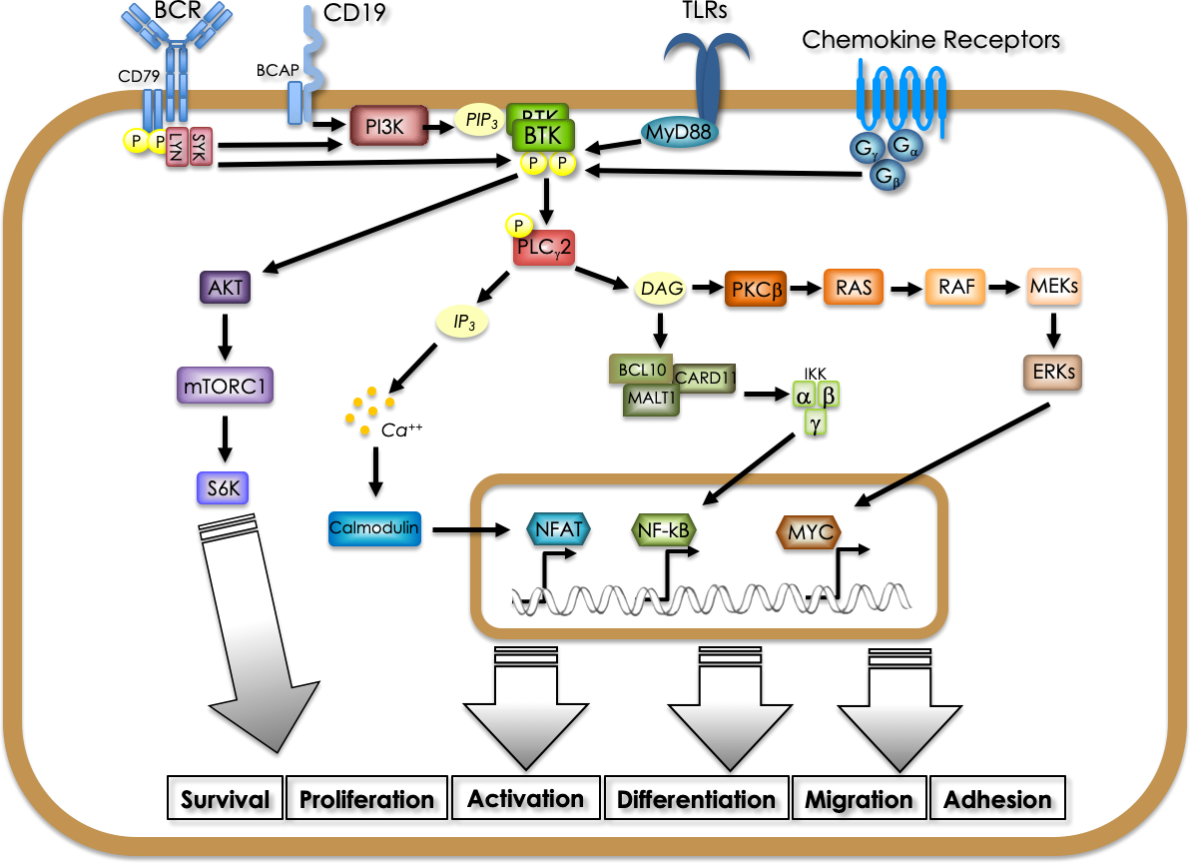
Signaling pathways involving BTK in B cells. BTK is activated downstream of different receptors (BCR, CD19, BAFFR, TLRs, chemokine receptors), being pivotal for relaying activation, proliferation, differentiation, survival, migration and adhesion signals. Different adaptors and kinases convey on BTK activation depending on the stimulated receptor. PI3K-generated PIP3 binding to the N-terminal PH domain allows membrane recruitment and phosphorylation-mediated activation. BTK-mediated phosphorylation activates PLCγ2 leading to IP3 and DAG generation which ultimately trigger NFAT-dependent transcription and RAS-MAPK and NF-kB pathway activation, respectively. In addition, BTK can activate directly the AKT-mTOR pathway.

Since the beginning, it has been recognized that BTK plays a pivotal role in B cell physiology. In fact, its activity was found necessary for relaying the signals triggered by the engagement of the B-cell receptor (BCR), thus being involved in the crucial steps of differentiation, proliferation, maturation and survival of B cells. In particular, upon engagement of the BCR by a specific antigen, LYN kinase is first recruited at the intracellular portion of the receptor, where it activates SYK that in turn phosphorylates PI3K, thus enabling PIP3 generation. PIP3 binding to BTK N-terminal PH-domain allows its recruitment (in a dimeric form) to the plasma membrane and its subsequent activating phosphorylation by Src-family kinases LYN or SYK on Y551. LYN and SYK also phosphorylate tyrosine residues in the cytoplasmic tail of the B-cell co-receptor CD19 and/or the adaptor protein B-cell PI3K adaptor (BCAP), which facilitates recruitment and activation of PI3K thus reinforcing the signaling that activates BTK. Autophosphorylation on Y223 allows full BTK activation that can then phosphorylate PLCγ2. The formation of this BCR signalosome (in which other components also participate) leads to the activation of multiple downstream signaling pathways (2). In fact, PLCγ2 *via* IP3 formation modulates Ca^++^ fluxes whereas *via* DAG formation activates PKCβ, that in turn signals onto the RAS/MAPK pathway and triggers NF-kB activation. In addition, BTK can activate AKT thus leading to mTORC1-mediated signaling (3). Beyond its pivotal role in transducing signals from the BCR, during the years BTK has been identified as a main player for transducing activation signals generated in B cells through the engagement of different receptors such as the Toll-Like Receptors (TLRs) and the chemokine receptors (4–6). Depending on the receptor different interactors and transducers have been shown to be involved in BTK-mediated signaling ultimately leading to downstream activation of NF-κB, AKT and MAPK-dependent pathways (3) (**Figure 1**). For example, in the case of TLRs their engagement by structurally conserved molecules derived from bacteria and viruses leads to the recruitment of the adaptor myeloid differentiation primary response 88 (MYD88), which then interacts directly with BTK. TLR signaling, beside the pathways mentioned above, also induces interferon regulatory factor 3 (IRF3) and all of them contribute to activation, proliferation, antibody secretion, class switch recombination and pro-inflammatory cytokine production in B cells. Chemokine receptors are seven transmembrane-spanning domain receptors that are coupled to intracellular hetero-trimeric G-protein (Gα, Gβ, and Gγ). When chemokines bind to the receptor a conformational change is induced resulting in dissociation of Gα and Gβy subunits which can directly bind BTK *via* the PH and TH domain, thus stimulating its activation. In addition, Gα and Gβγ subunits can independently activate PI3K, which results in the activation of BTK and subsequent signaling (3).

Since the initial recognition of BTK as a leading actor on the B cell scene, its role has then been explored in all aspects of immunity and nowadays BTK is considered a vital protein expressed by immunocompetent cells of both innate and adaptive immunity (6). In fact, the persistence of BTK-driven activated B cells is involved in several autoimmune and inflammatory diseases. In addition, BTK plays a key role in macrophages, in myeloid cells and in mast cells activation *via* the Fcγ receptors. A neutrophil-BTK-signalosome enhances neutrophil recruitment during inflammation (6) and the kinase positively regulates the large multi-protein complex known as NLRP3 inflammasome, whose activity has been reported to sustain important pathophysiological changes in a number of complex diseases (myocardial infarction, stroke, liver inflammation, type 2 diabetes, Alzheimer’s disease, Parkinson’s disease, sepsis) (7). In addition BTK activity is crucial in transmitting signals from platelet glycoprotein (GP)Ib/IX complex to allow platelet aggregation (3). It is now evident that, depending on the cell type, BTK can receive pleiotropic signals and relays to several signaling pathways, thus being crucial for many different biological processes.

## BTK isoforms and their expression in tumors

Given that BTK is a critical actor for B cell activation and proliferation, its overexpression and hyperactivation have been initially reported in acute lymphoblastic leukemias and plasmacytomas (8). To date BTK overexpression/hyperactivation has been shown in several B cell-derived malignancies such as chronic lymphocytic leukemia (CLL)/small lymphocytic lymphoma (SLL), mantle cell lymphoma (MCL), Waldenström’s Macroglobulinemia (WM), Marginal Zone Lymphoma (MZL), in the most common form of non-Hodgkin lymphoma i.e., activated B-cell diffuse large B-cell lymphoma (ABC-DLBCL), and in multiple myeloma (MM) (3). Notably, in the last decade it has progressively become evident that BTK is also expressed outside of the hematopoietic compartment, especially in some solid tumors. In addition, other isoforms have been discovered that are mainly expressed in solid tumors, and not in the immune cells. Remarkably, in all the isoforms the kinase domain is conserved allowing them to be equally inhibited by most of the BTK inhibitors (BTKis) actually available. The 77 kDa protein originally discovered is now referred to as BTK-A to distinguish it from the other isoforms.

### BTK-C

**Figure 2:**
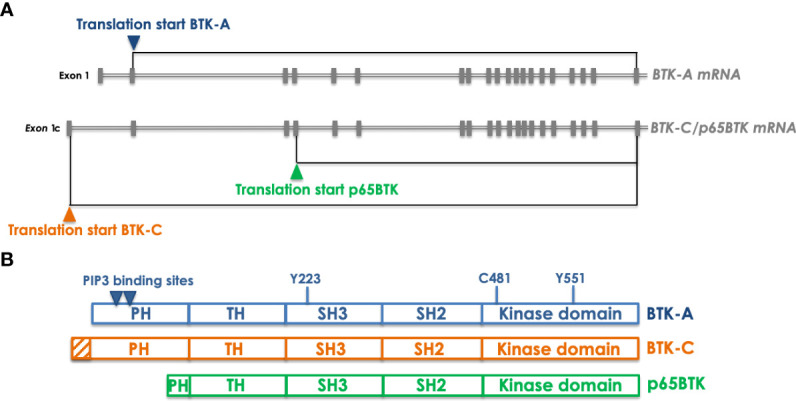
Transcripts encoding the different BTK isoforms and the corresponding proteins. **(A)** organization of the BTK-A- and BTK-C/p65BTK-encoding transcripts. The only variation between the two mRNAs is a different exon 1 which is uncoding in BTK-A transcript and encoding in the BTK-C one. In addition, the same mRNA can be translated also from an ATG located in the 4th exon thus giving raise to p65BTK. Gray boxes indicate exons. Translation starts are indicated by colored triangles. **(B)** Domain organization of the BTK isoforms. Compared to BTK-A BTK-C has an extended PH domain whereas p65BTK miss most of it. Indicated by triangles are the PIP3 binding sites that allow dimerization and membrane translocation. Y551and Y223 are the sites of phosphorylation that regulate BTK activation. C481 is the critical residue (gatekeeper) in the kinase domain targeted by most BTKis.

The first isoform, isolated from Conklin’s group, has been the so-called BTK-C, an 80 kDa protein translated from a messenger displaying an alternative first exon - compared to the BTK-A-encoding transcript – regulated by a different promoter (9). BTK-C mRNA gives rise to a protein with an extended N-terminal (**Figure 2**) and is expressed in approximately 15% of normal and tumor cells in prostate, bladder, lung squamous and breast tumor samples. Notably, its targeting in prostate and breast cancer cells impacts on cell survival by reducing proliferation and promoting cell cycle arrest - *via* downregulation of cyclin D1 – and apoptosis (10). In breast cancer cells BTK-C – as in the case of BTK-A – is activated by Src-family kinases and its inhibition results in decreased phosphorylation of known downstream effectors such as PLCγ2, AKT and ERKs (11). In prostate cancer cells overexpression of BTK-C is associated with elevated expression of genes with functions related to cell adhesion, cytoskeletal structure and the extracellular matrix, suggesting that BTK-C-regulated biological processes partially overlap with those affected by BTK-A, for which an important role has been demonstrated in adhesion and migration of B cells (12). On the whole, it seems that BTK-C in epithelial tumor cells is activated by the same molecular mechanisms and signals to the same downstream effectors as BTK-A does in B cells.

### p65BTK

A second BTK isoform originating from the translation of the same messenger encoding BTK-C has been independently identified and named p65BTK from its apparent molecular weight (13) (**Figure 2**). p65BTK translation starts from a non-canonical ATG located in the fourth exon and is regulated by an IRES- and HNRPK-dependent mechanism under the control of the MAPK pathway. Notably, p65BTK lacks most of the PH domain, necessary for PIP3 binding, allowing BTK translocation to the membrane and its activation. Structural studies demonstrated that the lack of the N-terminal increases the levels of spontaneous p65BTK activation, compared to BTK-A (14). In addition, the missing N-term is also the region where the interaction with several negative regulators occurs, suggesting that the lack of the first N-terminal 86 aminoacids might lead to an aberrantly expressed and activated protein. In fact, in colorectal carcinoma (CRC) samples p65BTK is abundantly expressed and correlates with active MAPKs. Moreover, at variance with the other isoforms, p65BTK has been demonstrated to be oncogenic and necessary for RAS-mediated transformation (13). Notably, in CRC its expression significantly increases with histological tumor grade (15) and stage III patients expressing the highest intensity of p65BTK in >80% cells have the worst prognosis in terms of disease-free survival and overall survival, indicating p65BTK as a potential prognostic marker (16). p65BTK targeting in drug-resistant p53-null CRC cells abolishes a 5-FU-elicited TGFB1 protective response and triggers E2F-dependent apoptosis. Moreover, adding BTKis to EGFR-targeting drugs induces significant cytotoxicity in EGFR inhibitors-resistant CRC cells (15). p65BTK is expressed in >50% of non-small cell lung cancer (NSCLC) samples, being its levels significantly higher in EGFR-wt samples, derived from patients not eligible for targeted therapy. Significantly, the use of BTKis overcomes resistance to chemotherapy and EGFR-directed inhibition also in NSCLC models and independently of EGFR, RAS or p53 mutation (17). Therefore, it seems that p65BTK is an important determinant of drug resistance, whose targeting would allow to bypass the inefficacy of conventional chemotherapy in presence of p53 loss/inactivation and of EGFR targeted therapy in presence of EGFR or RAS mutation. Notably, p53 loss/inactivation occurs in ~50% of all cancers (18), whereas RAS mutations are the second most frequent mutation occurring in ~20% of all cancers (reaching 50% in gastrointestinal and 70% in pancreatic cancers) (19). The repurposing of BTKis for the treatment of solid cancers might thus represent the way to re-sensitize otherwise non-responsive CRC and NSCLC tumors to standard chemotherapy and targeted therapy, thus offering renewed hope for drug-resistant cancer patients.

Moreover, p65BTK might be a prognostic indicator and an actionable target in glioblastoma and ovarian carcinoma. In fact, its expression occurs in 1/5 of glioblastoma (GBM) patients and is suggestive of worst prognosis for those with grade III gliomas; interestingly, 92.3% of BTK-positive patients’ samples show also co-expression of EGFR and p53 (indicative of mutated p53). Notably, BTK inhibition in patient-derived GBM stem cell lines significantly reduces metabolic activity and mitotic index and increases cell death (20). In ovarian cancer (OC) high p65BTK levels correlate with early relapse and worse progression-free survival and its targeting in *in vitro* and *ex-vivo* systems affects cell proliferation and survival (21).

Even though the signaling inputs and downstream effectors of p65BTK have been only partially identified this isoform is likely involved in different signaling pathways compared to the BTK-A and -C isoforms.

### BTK-A

Notably, also BTK-A has been reported to be highly expressed in different solid tumors such as neuroblastoma, glioma, oesophageal, gastric and bladder cancers (22–29). However, in several cases BTK’s nature has not been directly investigated and given that most of the experiments have been performed using BTKis – effective on all the isoforms - the specific isoform expressed by the abovementioned tumors is not certain. Despite that, the experiments performed on those tumor models clearly indicate that BTKis decrease the expression of stemness markers, reduce proliferation and clonogenicity, impair migration and invasion capabilities and induce variable levels of apoptosis, that can be significantly increased when used in combination with chemotherapy. In addition, in some *in vivo* models the anti-tumor effect of BTKis has been ascribed to the action on the immune and inflammatory cells present in the tumor micro-environment. For example, in a pancreatic adenocarcinoma model BTK has been shown to regulate B-cell and macrophage-mediated T-cell suppression and its inhibition restored T cell-dependent antitumor immune responses to inhibit tumor growth and improves responsiveness to chemotherapy (30). Instead, in a breast cancer model ibrutinib inhibited tumor development and metastasis by promoting the development of mature dendritic cells from myeloid-derived suppressor cells (31).

## Targeting BTK

The initial finding that BTK dysregulation occurs in pathologies characterized by excessive B cell proliferation/activation paved the way for the development of specific inhibitors. The very first reported rationally-designed BTKi was LFM-A13 (32) which was demonstrated to chemosensitize and promote apoptosis both *in vitro* and *in vivo* of chemotherapy-resistant B leukemic cells (33). Despite the proof-of-concept obtained using this molecule it did not progress to the clinic. At the beginning of the last decade the first BTK irreversible inhibitor PCI-32765 (then renamed ibrutinib) was shown to block B-cell activation and be efficacious in models of autoimmune disease and B-cell malignancies (34). In July 2013 a new drug application was submitted to the US Food and Drug Administration (FDA) – through the new Breakthrough Therapy Designation pathway - for using ibrutinib in relapsed patients affected by CLL/SLL and MCL. Between November 2013 and February 2014 a fast-track approval was given for the treatment of both B-cell malignancies and since then ibrutinib use has been expanded. More specifically, today Ibrutinib is given to pretreated adults with MCL, previously treated, or untreated CLL/SLL and WM patients. In addition, ibrutinib is also used as a first-line therapy in CLL and SLL in combination with obinutuzumab (anti-CD20), thus providing an alternative to frontline treatment with chemotherapy. FDA has also approved ibrutinib for previously treated MZL patients and chronic graft-versus-host disease (cGVHD) (https://www.fda.gov). In Europe the European Medicines Agency (EMA) approved ibrutinib-based therapy for refractory and pre-treated MCL and WM patients and for previously treated and untreated CLL patients. In the latter case bendamustine and rituximab or obinutuzumab or rituximab can be given in association with the BTK inhibitor (https://www.ema.europa.eu).

In just a few years, BTK targeting has become one of the most promising and pursued approaches in hematological oncology and several other inhibitors entered the scene (**Table 1**). Recently, acalabrutinib received FDA and EMA approval for the therapy of untreated or refractory CLL/SLL and only FDA approved for pretreated MCL patients. The US agency also granted fast-track approval for zanubrutinib for treating MCL whereas EMA approved its use in WM patients who have not been treated before and who cannot receive chemo-immunotherapy or in patients who have received at least one prior therapy (https://www.fda.gov/; https://www.ema.europa.eu). Moreover, in China the National Medical Products Administration (NMPA) approved its use for treating CLL/SLL and pretreated MCL patients and recently granted a conditional approval for the treatment of adult patients with WM who have previously received at least 1 therapy (http://english.nmpa.gov.cn). Finally, tirabrutinib has been approved in Japan for the treatment of recurrent or refractory primary central nervous system lymphoma, WM and lymphoplasmacytic lymphoma (https://www.pmda.go.jp). In addition, numerous others BTKis are currently in clinical trials (16 as of April 2022, clinicaltrials.gov, see **Table 2**).

**Table 1:** BTKis already approved.

	FDA	EMA	NMPA	PMDA
***ibrutinib***	pretreated MCL previously treated or untreated CLL/SLL (± obinutuzumab) WM MZL cGVHD	refractory and pre-treated MCL and WM previously treated and untreated CLL (± bendamustine and rituximab or obinutuzumab or rituximab)		
***acalabrutinib***	untreated or refractory CLL/SLL pretreated MCL	untreated or refractory CLL/SLL		
***zanubrutinib***	MCL	previously treated or untreated WM not eligible for chemo-immunotherapy	CLL/SLL and pretreated MCL conditional approval for previously treated WM	
***tirabrutinib***				recurrent or refractory primary central nervous system lymphoma, WM and lymphoplasmacytic lymphoma

FDA, Food and Drug Administration; EMA, European Medicines Agency; NMPA, National Medical Products Administration; PMDA, Pharmaceuticals and Medical Devices Agency; MCL, mantle cell lymphoma; CLL/SLL, chronic lymphocytic leukemia/small lymphocytic lymphoma; WM, Macroglobulinemia macroglobulinemia; MZL, marginal zone lymphoma; cGVHD, chronic graft-versus-host-disease.

**Table 2:** BTKis in clinical trial (as of April 2022, clinicaltrials.gov).

Inhibitor	Company
Fenebrutinib	Roche
Branebrutinib	Bristol-Myers Squibb
Evobrutinib	Merck KGaA
Pirtobrutinib	Ely Lilly
Remibrutinib	Novartis
Tolebrutinib	Sanofi
Rilzabrutinib	Principia Biopharma, (a Sanofi company)
Vecabrutinib	Sunesis Pharmaceuticals
Orelabrutinib	Beijing Inno Care Pharma Tec Co
TG-1701	TG Therapeutics
BIIB091	Biogen
NX-2127	Nurix Therapeutics
WXHS-12	Zhejiang DTRM Biopharma
SHR1459	Jiangsu HengRui Medicine Co
SN1011	SinoMab Pty Ltd
HZ-A-018	Hangzhou Hezheng Pharmaceutical

One of the main problems with ibrutinib – ultimately leading to the discontinuation of the therapy in 30% of patients - are the adverse effects due to the off-target effects generated by its mechanism of action i.e., the binding of the Cys-481 residue in the kinase domain of the enzyme. In fact, the targeted Cys residue is shared by few other kinases, among them other members of the TEC family and all the members of the EGFR family (35). For example, binding to both BTK and TEC in platelets is related to bleedings whereas atrial fibrillation may ensue as a result of inhibiting TEC in cardiomyocytes. In addition, cardiomyocyte dysfunction and reduced heart contractile efficiency are potentially related to ERBB2 inhibition. Finally, ibrutinib may also tethers reversibly SRC-family regulatory kinases whose inhibition can affect macrophages and platelets (expressing both BTK and SRC-family kinases) (36).

Another important issue is the resistance to the inhibitor that can develop over time - especially in MCL and high-risk CLL patients – due to the occurrence of a mutation of the gatekeeper residue Cys-481 (37). Second-generation BTKis (acalabrutinib, zanubrutinib and tirabrutinib) are highly selective with no cross-reactivity with EGFR family members, thus showing limited toxicity and being more potent than ibrutinib. However, they bind the same Cys-481 residue and resistance has been reported in patients with concomitant Cys-481 mutation (37). Novel third-generation BTK inhibitors and proteolysis-targeting chimeras (PROTACs) can effectively target both wild type and mutant BTK, and are currently in the early phase of clinical trials (38). In particular, non-covalent inhibitors, such as fenebrutinib, do not interact with the Cys-481 residue and thus they can still bind - and inhibit - mutants where Cys-481 is changed in Ser or Arg (37), the two most frequent mutation in patients (38). For the next generation BTKis another useful approach, allowing an ever increasing and more stringent specificity, will be the identification and/or synthesis of allosteric inhibitors.

## Conclusions and perspectives

**Figure 3:**
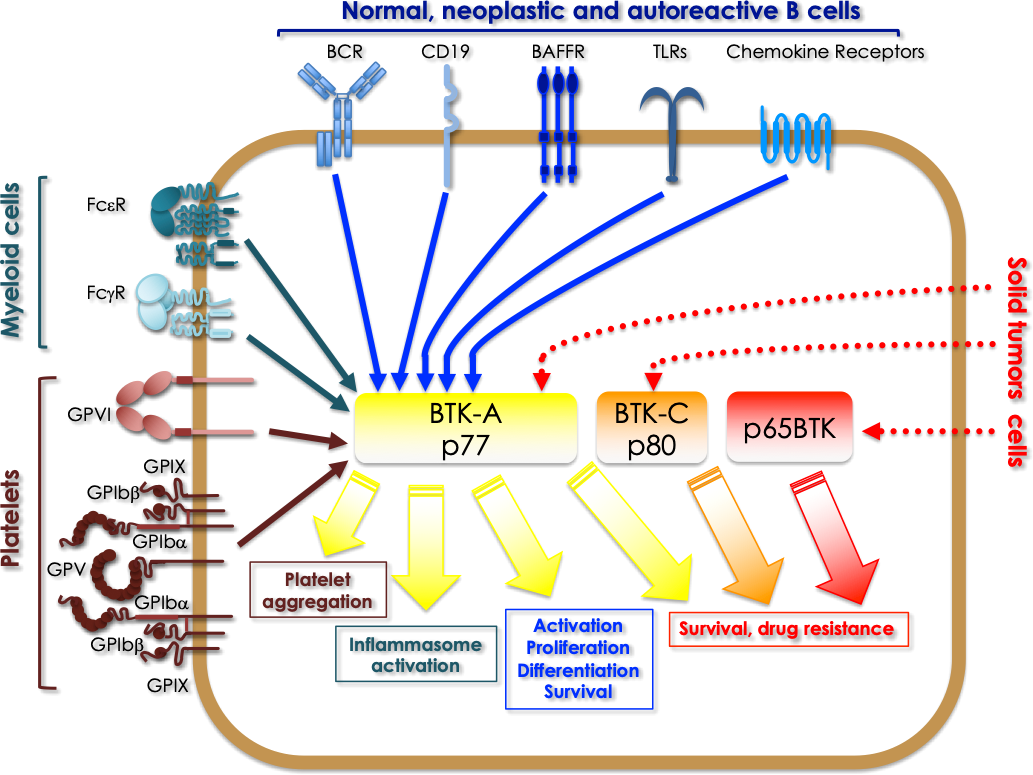
Overview of the signaling pathways where BTK isoforms are involved in different types of cells. BTK-A activation downstream of a series of receptors expressed in normal, neoplastic and autoreactive B cells (BCR, CD19, BAFFR, TLRs, chemokine receptors), regulates activation, proliferation, differentiation and survival signals. In myeloid cells the same isoform is activated by FcεR and FcγR and TLRs, which is crucial for the activation of the inflammasome. In platelets BTK-A activation is necessary for platelets aggregation triggered by the adhesive receptors. Finally, in some solid tumors, BTK-A pharmacological targeting has revealed its role in promoting the survival of cancer cells. A role for sustaining the survival of breast and prostate cancer cells has been demonstrated for the 80 kDa BTK-C isoform. Targeting p65BTK has proved to overcome the resistance of colon and non-small cell lung cancer cells to chemo- and targeted therapy, besides being a prognostic factor and an actionable target in glioblastoma and ovarian cancer.

In summary, in the last decade, it has become evident that not only BTK is central to several signaling pathways, relaying many receptors with diverse downstream effectors in bone marrow-derived cells but that – expressed in different isoforms - is also pivotal for many tumor cells (**Figure 3**). Notably, reduction of proliferation, of the clonogenic potential and of viability are common effects induced by BTKis in almost all solid tumor models studied - independently of which BTK isoform is expressed - thus indicating for all BTK isoforms a conserved role in cell survival. Based on this, it is easy to predict that the studies about re-purposing of BTKis for the treatment of solid cancers will expand extensively in the next decade, thus creating new opportunities to target this kinase for therapeutic benefit. Notably, to better repurpose the existing BTKis and those in clinical trials, an essential challenge will be to define the biological functions of each isoform and to identify the relevant signaling pathways in which each of them is involved. In fact, given that - on the long period - resistance to tyrosine kinase inhibitors almost inevitably occurs - mainly due to rewiring of parallel and redundant pathways (39–41) -, it is essential to understand which circuits must be concomitantly - parallelly or vertically – inhibited to prevent this occurrence. In addition, isoform-specific antibodies need to be developed to identify which isoform is expressed in which tumor and to ultimately develop appropriate diagnostic tools for identifying the patients eligible for BTKis-based therapy. At the moment, also very promising are the initial experimental evidence indicating that the addition of BTKis overcome resistance to chemo- and targeted therapy. Accordingly, it will be of paramount importance to identify which drug combinations are the most effective and for which tumors.

In conclusion, in the last decade, several evidences indicate that BTK, in all its forms, is the new kid on the (oncology) block, onto which a special attention will have to be payed, besides the “usual suspects” - EGFR, BRAF, MEKs - whose diagnostic investigation and inhibition has already become a clinical standard. Hopefully, in the next decade a better understanding of BTK(s) role(s) in cancer(s) will be reached, so that BTKis-based therapy will take center stage in the era of personalized medicine for treating cancer.

## Author contributions

All authors listed have made a substantial, direct, and intellectual contribution to the work, and approved it for publication.

## Conflict of interest

The authors declare that the research was conducted in the absence of any commercial or financial relationships that could be construed as a potential conflict of interest.

## Publisher’s note

All claims expressed in this article are solely those of the authors and do not necessarily represent those of their affiliated organizations, or those of the publisher, the editors and the reviewers. Any product that may be evaluated in this article, or claim that may be made by its manufacturer, is not guaranteed or endorsed by the publisher.
